# A Trajectory Tracking Control Based on a Terminal Sliding Mode for a Compliant Robot with Nonlinear Stiffness Joints

**DOI:** 10.3390/mi13030409

**Published:** 2022-03-04

**Authors:** Zhibin Song, Tianyu Ma, Keke Qi, Emmanouil Spyrakos-Papastavridis, Songyuan Zhang, Rongjie Kang

**Affiliations:** 1 Key Laboratory of Mechanism Theory and Equipment Design of Ministry of Education, Tianjin University, Tianjin 300072, China; matianyu@tju.edu.cn (T.M.); 2019201094@tju.edu.cn (K.Q.); rjkang@tju.edu.cn (R.K.); 2 School of Mechanical Engineering, Tianjin University, Tianjin 300072, China; 3 Centre for Robotics Research, Department of Engineering, King’s College, London WC2R 2LS, UK; emmanouil.spyrakos@kcl.ac.uk; 4 School of Mechatronics Engineering, Harbin Institute of Technology, Harbin 150001, China

**Keywords:** compliant robot, terminal sliding mode, Lyapunov stability, trajectory tracking

## Abstract

A nonlinear stiffness actuator (NSA) can achieve high torque/force resolution in the low stiffness range and high bandwidth in the high stiffness range. However, for the NSA, due to the imperfect performance of the elastic mechanical component such as friction, hysteresis, and unmeasurable energy consumption caused by former factors, it is more difficult to achieve accurate position control compared to the rigid actuator. Moreover, for a compliant robot with multiple degree of freedoms (DOFs) driven by NSAs, the influence of every NSA on the trajectory of the end effector is different and even coupled. Therefore, it is a challenge to implement precise trajectory control on a robot driven by such NSAs. In this paper, a control algorithm based on the Terminal Sliding Mode (TSM) approach is proposed to control the end effector trajectory of the compliant robot with multiple DOFs driven by NSAs. This control algorithm reduces the coupling of the driving torque, and mitigates the influence of parametric variation. The closed-loop system’s finite time convergence and stability are mathematically established via the Lyapunov stability theory. Moreover, under the same experimental conditions, by the comparison between the Proportion Differentiation (PD) controller and the controller using TSM method, the algorithm’s efficacy is experimentally verified on the developed compliant robot. The results show that the trajectory tracking is more accurate for the controller using the TSM method compared to the PD controller.

## 1. Introduction

Robots with flexible joints are a benefit to industry, rehabilitation, aviation, and marine exploration for their good interactive performance and security. Flexibility is usually divided into active flexibility and passive flexibility [1]. Active flexibility implies that the robot is rendered flexible through the control algorithm, despite the fact that its main body structure may still be rigid. Passive flexibility is introduced into the robot’s design, either through physically compliant actuators, or via soft links. The structure of the elastic component is typically used to make the joint output display a predetermined, desirable flexible behavior. Different from series elastic actuators with constant stiffness springs and variable stiffness actuators with extra motor to adjust stiffness [2,3], the nonlinear stiffness actuator (NSA) was proposed in our group to improve its perceptivity and responsiveness to external loads, where the positional shape and the stiffness curve of the elastic element can be specifically designed [4,5]. However, addition of the elastic element might exacerbate the control problem. For example, friction, hysteresis and part of the energy consumed by the robot’s nonlinear elastic components may not be accurately detected in real time. Also, the change of the moment of inertia of some components of robots during motion is not accurately calculated. These problems will affect the robot’s trajectory tracking control accuracy [6,7,8].

With the advent of compliant robots, the development of appropriate control methods for these systems is a topic that has attracted widespread attention from many experts and scholars. Even though classic PD controllers appear to be a natural choice for flexible-joint robot control, these schemes tend to display undesirable amounts of overshoot and performance degradation in the presence of external disturbances [9]. To deal with the disturbance rejection problem, Han proposed an active disturbance suppression technology based on classic PD control, which can effectively control systems with uncertainty and external disturbances [10]. However, the theoretical analysis revolving around the Auto Disturbance Rejection Controller (ADRC)’s linear, non-smooth feedback structure is rather complex, as it requires meticulous adjustments of an array of control parameters. Spong proposed simplification of the flexible joint dynamics, via the consideration of two nonlinear, second-order differential equations that are coupled to each other through linear springs. The two resulting subsystems were then controlled by means of integral flow and perturbation theory [11].

Active impedance control [12], whose operation relies upon linear spring-damper models, is deemed a conventional control strategy for compliant robots. Impedance control can enable seamless interaction between a robotic system and its external environment, whilst also permitting the execution of desired trajectories (during free motion). There are many other scholars who have conducted in-depth research on this issue. For example, Albu-Schäffer et al. conducted research on impedance control for flexible-joint robots, which they further advanced via the introduction of passivity-based analyses, torque control interfaces, and unconstrained active stiffness gain selection [13]. Sira-Ramirez and Spong designed a sliding mode controller [14], and Zeman et al. proposed the usage of neural networks to compensate for system parameter uncertainties [15]. Dong-Dong Zheng et al. propose a neural-network-based system identification and control study for the nonlinearity of flexible joints [16,17]. In addition, many researchers adopted neural networks, incremental learning, and other methods to control compliant robots [18,19,20,21,22].

The disturbance observer proposed by Ohnishi in 1987 can be used to estimate the system perturbations that are difficult to measure. The external disturbance is estimated through the input signal magnitude, and the inner loop feedback value [23]. Hence, this observed compensation value is then filtered and appended to the control signal, in order to cancel out the actual disturbances. With the increase of the device order, the large phase lag causes the system to be underdamped and unstable. As a result of this, although these models can realize torque and position control, they are highly dependent on the Lagrangian dynamics model. The number of required calculations scales with the robot’s degrees of freedom, and this complexity is further compounded when accounting for external disturbances.

In recent years, adaptive control, neural network control and other methods to reduce the impact of parameter uncertainty have shown strong robustness [24,25,26,27]. C. Sun proposes an adaptive neural network control method [28], but in the process of practical application, the parameter design is complex. The fuzzy controller shows good performance in these kinds of problem, but its controller design is also difficult. On the contrary, a sliding mode control is developed rapidly for its simple structure and good applicability [29,30]. Sliding mode control can deal with complex nonlinear and uncertain problems and has strong robustness [31]. Through the structure design, the sensitivity of disturbance and parameters can be reduced, and the robot system model can be more inclusive.

In this paper, to improve the trajectory tracking control of the robot with NSA, a control algorithm based on terminal sliding mode (TSM) was designed. The end trajectory tracking control of the compliant robot in this paper reduces the coupling of torque and the influence of parameter changes, and can deal with the irresistible interference of the system. The finite time convergence and stability of the control system are verified. The TSM algorithm is compared with the traditional PD optimal parameter controller under the same experimental conditions, which verifies the feasibility of the TSM algorithm.

This paper is organized as follows; the flexible-joint robot prototype’s dynamical model is introduced in Section 2. Subsequently, the design of the Terminal Sliding Mode (TSM) controller is introduced in Section 3, while a stability analysis based on Lyapunov theory is presented in Section 4. Furthermore, experimental results are presented in Section 5, and a conclusion is provided in Section 6.

## 2. Methodology

### 2.1. Brief Introduction of the Developed Compliant Robot with NSAs

**Figure 1:**
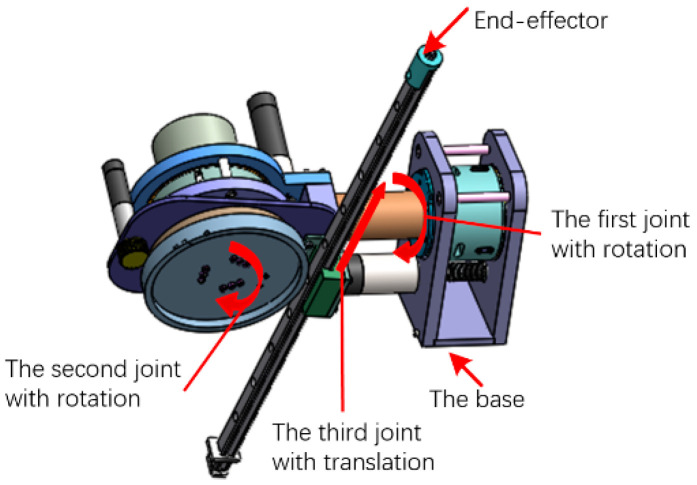
The proposed compliant robot with three DoFs.

The concept of NSA was proposed based on a common law of physical contact in natural phenomenon [4]. The stiffness of the NSA varies continuously and adaptatively with the external torque. To be specific, when the external torque is small, the NSA performs with a low stiffness; while when the external torque is large, the NSA performs with a high stiffness. This property makes the NSA achieve the high torque/force resolution in the low stiffness range and the high bandwidth in the high stiffness range. We not only fabricated a prototype of such actuator, we also built a 3-DOF compliant robot (diagrammed in Figure 1) with nonlinear stiffness joints in our laboratory. The details of NSA are presented in [4,32]. Figure 1 shows the 3D models of the developed 3-DOF robot including two rotations and one translation. The inner structure of the robot and working principle are therefore omitted in this work.

Although the rotational position of single NSA has been controlled well by building a new model of hysteresis and Proportion Integration Differentiation (PID) with optimized parameters, it is still very difficult to implement accurate trajectory tracking of the end-effector of the developed robot with several NSAs since the coupling influence of each NSA and friction depends on the robot’s configuration. Based on the kinematics, the angles of three constituent joints can be calculated via inverse kinematics, in accordance with the desired trajectory. The three joints are then controlled in accordance with a time-based scheme.

### 2.2. Dynamic Model

**Figure 2:**
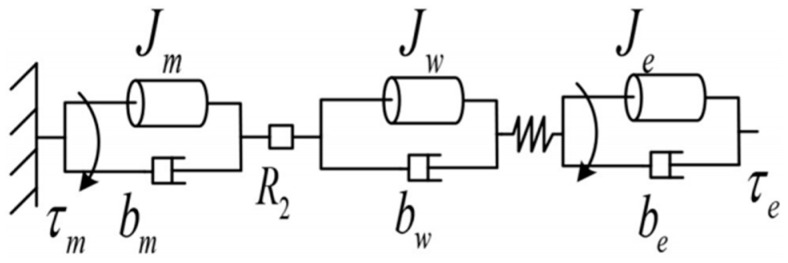
Schematic diagram of the NSA.

The nonlinear stiffness drive model, according to [18], includes the power system, transmission system, elastic structure, and external load. The power system is the motor combination, which mainly includes the motor rotor and the gear reducer. The equivalent moment of inertia of the motor combination can be obtained from the dynamics model of the motor combination. The dynamic equations describing the motor rotor, reducer, and output shaft may be represented as follows according to Figure 2:

(1) $$J_{r}{\ddot{\theta }}_{r}+b_{r}{\dot{\theta }}_{r}=\tau_{m}-\tau_{r}$$

(2) $$\frac{{\ddot{\theta }}_{r}}{{\ddot{\theta }}_{g}}=\frac{{\dot{\theta }}_{r}}{{\dot{\theta }}_{g}}=\frac{\theta_{r}}{\theta_{g}}=R_{1}$$

(3) $$J_{g}{\ddot{\theta }}_{g}+b_{g}{\dot{\theta }}_{g}=R_{1}\tau_{r}-\tau_{g}$$

(4) $$\frac{{\ddot{\theta }}_{g}}{{\ddot{\theta }}_{w}}=\frac{{\dot{\theta }}_{g}}{{\dot{\theta }}_{w}}=\frac{\theta_{g}}{\theta_{w}}=R_{2}$$

(5) $$J_{w}{\ddot{\theta }}_{w}+b_{w}{\dot{\theta }}_{w}=R_{2}\tau_{g}-\tau_{k}$$

where $J_{r}$ and $b_{r}$ are the moment of inertia and damping of the motor rotor, respectively; ${\dot{\theta }}_{r}$ and ${\ddot{\theta }}_{r}$ are the angular velocity and angular acceleration of the motor rotor, respectively; $\tau_{m}$ is the torque generated by the motor rotor; $\tau_{r}$ is the torque output by the motor rotor; $J_{g}$ and $b_{g}$ are the moment of inertia and damping of the motor reducer, respectively; ${\ddot{\theta }}_{g}$ and ${\dot{\theta }}_{g}$ are the angular velocity and angular acceleration of the motor reducer, respectively; $R_{1}$ is the reduction ratio; $\tau_{g}$ is the torque output by the motor reducer; ${\ddot{\theta }}_{w}$ and ${\dot{\theta }}_{w}$ are the angular velocity and angular acceleration of the nonlinear stiffness drive, outer drum’s output shaft, respectively; $R_{2}$ is the reduction ratio of the wire drive. Solving Equations (1)–(5) simultaneously can obtain:

(6) $$\left(J_{r}+\frac{1}{R_{1}{}^{2}}J_{g}+\frac{J_{w}}{R_{1}{}^{2}R_{2}{}^{2}}\right){\ddot{\theta }}_{r}+\left(b_{r}+\frac{1}{R_{1}{}^{2}}b_{g}+\frac{b_{w}}{R_{1}{}^{2}R_{2}{}^{2}}\right){\dot{\theta }}_{r}=\tau_{m}-\frac{\tau_{k}}{R_{1}R_{2}}$$

Then the equivalent dynamic Equation of the motor assembly and the elastic part is:

(7) $$J_{eq}{\ddot{\theta }}_{r}+b_{eq}{\dot{\theta }}_{r}=\tau_{m}-\frac{\tau_{k}}{R_{1}R_{2}}$$

where $J_{eq}=J_{r}+\frac{1}{R_{1}{}^{2}}J_{g}+\frac{J_{w}}{R_{1}{}^{2}R_{2}{}^{2}}$ is the actuator equivalent inertia, and $b_{eq}=b_{r}+\frac{1}{R_{1}{}^{2}}b_{g}+\frac{b_{w}}{R_{1}{}^{2}R_{2}{}^{2}}$ is the actuator equivalent damping.

The dynamic Equation of the outer cylinder part is:

(8) $$J_{e}{\ddot{\theta }}_{e}+b_{e}{\dot{\theta }}_{e}=\tau_{k}-\tau_{e}$$

where $J_{e}$ and $b_{e}$ are the moment of inertia and damping of the external load, respectively; $\tau_{e}$ is the output torque of the drive; ${\ddot{\theta }}_{e}$ and ${\dot{\theta }}_{e}$ are the angular velocity and angular acceleration of the external load, respectively.

It can be seen from the above formula that the dynamic Equation from the motor to the output shaft (without considering the external torque input) is:

(9) $$J_{e}{\ddot{\theta }}_{e}+b_{e}{\dot{\theta }}_{e}=R_{1}R_{2}(\tau_{m}-J_{eq}{\ddot{\theta }}_{r}-b_{eq}{\dot{\theta }}_{r})$$

## 3. TSM Controller

**Figure 3:**
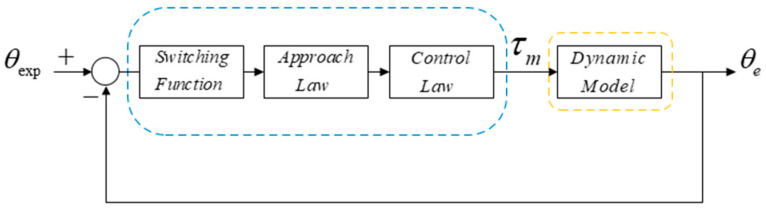
The schematic of TSM controller.

The terminal sliding mode controller is a kind of robust nonlinear controller which has an unfixed structure and avoids the influence of coupling parameters and disturbance, therefore, it is adopted as a main controller for our compliant with NSAs. In this paper, we designed a TSM mode for our robot and it is introduced as follows. It consists of three parts: the design of the switching function, whose purpose is to acquire a sliding mode surface that nullifies the position tracking error, preserves closed-loop stability, and achieves heightened control performance; the design of the approach law, such that it can rapidly reach the set interface, and ensure stability of the control system without chattering; the design of a control law that generates an input, which is capable of ensuring a stable output. The purpose of the terminal-based sliding mode control scheme designed is to achieve accurate end-effector trajectory tracking. According to this target task, the designed terminal sliding mode controller is shown in Figure 3, where it is assumed that the system’s input is the desired end-effector trajectory. The sliding mode control law produces the torque required by the drive, which is then transmitted to the robotic system that in turn realizes the desired end-effector trajectory.

The drive dynamics (Equation (9)) can be rewritten in the following manner:

(10) $$\tau_{m}=\frac{1}{R_{1}R_{2}}\left(J_{e}{\ddot{\theta }}_{e}+b_{e}{\dot{\theta }}_{e}\right)+J_{eq}{\ddot{\theta }}_{r}+b_{eq}{\dot{\theta }}_{r}$$

The design procedure leading to creation of the terminal sliding-mode controller can be described as follows:

By defining the desired position of the compliant robot system as $\theta_{\exp}$, and the end effector trajectory output error as $e=\theta_{\exp}-\theta_{e}$, the output error derivative variable can then be obtained as follows:

(11) $$\dot{e}={\dot{\theta }}_{\exp}-{\dot{\theta }}_{e}$$

Computing Equation (11)’s time derivative, and substituting it into Equation (10), yields the following expression:

(12) $$\dot{e}=\frac{1}{J_{e}}\left[R_{1}R_{2}\tau_{m}-b_{e}{\dot{\theta }}_{e}-R_{1}R_{2}\left(J_{eq}{\ddot{\theta }}_{r}+b_{eq}{\dot{\theta }}_{r}\right)\right]-{\ddot{\theta }}_{\exp}$$

Based on the robotic system’s error that is defined above, a switching function can then be designed (namely, the terminal sliding mode surface), as follows:

(13) $$s=\alpha e+\beta \dot{e}+\chi \mathrm{sgn}{\left(\dot{e}\right)}^{\gamma }$$

where $\mathrm{sgn}{\left(\dot{e}\right)}^{\gamma }={\left|\dot{e}\right|}^{r}\mathrm{sgn}\left(\dot{e}\right)$, α, β and χ are the system parameters for the switching function, which are all greater than zero, while 1<γ<2. According to the designed switching function, in order to make the system converge within a finite time, the design control rate is defined as:

(14) $$\dot{s}=-\tilde{\alpha }s-\tilde{\beta }\mathrm{sgn}{\left(s\right)}^{\rho }$$

where $\tilde{\alpha }$, $\tilde{\beta }$ and ρ are the approach law’s design parameters, which are positive constants. The system’s control law may then be designed as follows:

(15) $$\tau_{m}=\frac{1}{R_{1}R_{2}}\left(J_{e}\left(-\tilde{\alpha }s-\tilde{\beta }\mathrm{sgn}{\left(s\right)}^{\rho }+{\ddot{\theta }}_{\exp}-\frac{\alpha \dot{e}}{\beta +\chi \gamma {\left|\dot{e}\right|}^{\gamma -1}}\right)+b_{e}{\dot{\theta }}_{e}\right)+J_{eq}{\ddot{\theta }}_{r}+b_{eq}{\dot{\theta }}_{r}$$

In the actual application process, the terminal sliding mode controller, whose target task is the end-effector position tracking control, will encounter discontinuity problems due to the approach law’s structure. In order to further suppress chattering effects, this paper adopts a type of saturation function. The saturation function’s definition is the following:

(16) $$sat(s)=\{\begin{array}{c} \mathrm{sgn}(\frac{s}{\delta }),\left|\frac{s}{\delta }\right|>1\\ \frac{s}{\delta },\left|\frac{s}{\delta }\right|\leq 1 \end{array}$$

where δ>0. In its current form, the trajectory tracking controller design based on terminal sliding mode control, is tailored to compliant robots with nonlinear stiffness actuators, small parametric changes.

## 4. Lyapunov Stability Analysis

Lyapunov stability theory is an effective method for analyzing control system stability. This paper provides a stability proof, for the purpose of corroborating the mathematical soundness of the proposed controller. In order to prove stability and convergence of the designed closed-loop system, the lemma described in [26] is introduced.

**Lemma** **1.** *For any continuous, non-Lipschitz differential equation defined in the real field, an accompanying Lyapunov function’s derivative must satisfy the relationship:*

(17) $$\dot{V}\left(x\right)+\alpha_{1}V\left(x\right)+\alpha_{2}V^{\gamma }\left(x\right)\leq 0$$

That is, if the Lyapunov derivative function is negative semidefinite, then its convergence time can be obtained as follows:

(18) $$T_{finite-time}\leq \frac{1}{\alpha_{1}\left(1-r\right)}\ln\frac{\alpha_{1}V^{1-\gamma }\left(x_{o}\right)+\alpha_{2}}{\alpha_{2}}$$

In accordance with this lemma, it is known that for any continuous non-Lipschitz function, the system is globally finite time stable, that is, for any given initial condition, the system state converges for a finite time and always remains stable. Additionally, the convergence time is related to the initial state value.

In order to prove stability of the closed-loop system considered herein, a candidate Lyapunov function is constructed as per the designed terminal sliding mode control system, in the following manner:

(19) $$V=\frac{1}{2}s^{2}$$

Computing the derivative of the proposed Lyapunov function, yields the expression:

(20) $$\dot{V}=s\dot{s}=s\left(-\tilde{\alpha }s-\tilde{\beta }\mathrm{sgn}{\left(s\right)}^{\rho }\right)=-\tilde{\alpha }s^{2}-\tilde{\beta }\mathrm{sgn}{\left(s\right)}^{\rho }s\leq 0$$

Substituting Equation (19) into Equation (20), produces:

(21) $$\dot{V}=-\tilde{\alpha }s^{2}-\tilde{\beta }\mathrm{sgn}{\left(s\right)}^{\rho }s=-2\tilde{\alpha }V-2\tilde{\beta }{\left|V\right|}^{\frac{1+\rho }{2}}$$

Since $\dot{V}\leq 0$, Equation (21) can be rewritten as follows:

(22) $$\dot{V}+2\tilde{\beta }{\left|V\right|}^{\frac{1+\rho }{2}}+2\tilde{\alpha }V\leq 0$$

According to the lemma, the convergence time can be obtained via the expression:

(23) $$T_{finite-time}\leq \frac{1}{2\tilde{\beta }\left(1-\frac{1+\rho }{2}\right)}\ln\frac{2\tilde{\beta }V^{1-\frac{1+\rho }{2}}\left(x_{o}\right)+2\tilde{\alpha }}{2\tilde{\alpha }}$$

The above analysis reveals that the designed control system can converge within a finite time under given initial conditions, i.e., the control system is stable.

The control system designed in this paper should offer a certain level of robustness to slight variations of the moment of inertia, energy losses of the elastic component, and interference. To verify the aforesaid feature, a control system robustness analysis is carried out.

Considering the system’s kinetic energy evolution, and slight variation caused by the moment of inertia, the dynamic equation can be rewritten as:

(24) $$\tau_{m}=\frac{1}{R_{1}R_{2}}\left(\left(J_{e}+\Delta J_{e}\right){\ddot{\theta }}_{e}+\left(b_{e}+\Delta b_{e}\right){\dot{\theta }}_{e}\right)+J_{eq}{\ddot{\theta }}_{r}+b_{eq}{\dot{\theta }}_{r}+\tau_{dis}+\tau_{con}$$

where $\Delta J_{e}$ and $\Delta b_{e}$ represent slight changes in moment of inertia and damping; $\tau_{dis}$ and $\tau_{con}$ represent the energy loss and external disturbance caused by the nonlinear elastic component’s deformation, which satisfy the bounded convergence, then Equation (24) can be simplified as:

(25) $$\tau_{m}=\frac{1}{R_{1}R_{2}}\left(J_{e}{\ddot{\theta }}_{e}+b_{e}{\dot{\theta }}_{e}\right)+J_{eq}{\ddot{\theta }}_{r}+b_{eq}{\dot{\theta }}_{r}+D$$

where *D* represents a combination of the slight changes of moment of inertia and damping, the energy loss term caused by the deformation of the nonlinear elastic element, and the total torque change caused by the external disturbance term. Usually, in the physical robotic system, disturbance of system parameters, and external interference, are inevitable. Equation (25) can therefore be rewritten as:

(26) $${\ddot{\theta }}_{e}=\frac{R_{1}R_{2}\left(\tau_{m}-J_{eq}{\ddot{\theta }}_{r}-b_{eq}{\dot{\theta }}_{r}-D\right)-b_{e}{\dot{\theta }}_{e}}{J_{e}}$$

Considering external disturbance of the robotic system, and moment of inertia losses, the derivative of the switching function is expressed as follows:

(27) $$\dot{s}=\alpha \dot{e}+\left(\beta +\chi \gamma \mathrm{sgn}{\left(\dot{e}\right)}^{\gamma -1}\right)\ddot{e}=\alpha \dot{e}+\left(\beta +\chi \gamma \mathrm{sgn}{\left(\dot{e}\right)}^{\gamma -1}\right)\left(\frac{R_{1}R_{2}\left(\tau_{m}-J_{eq}{\ddot{\theta }}_{r}-b_{eq}{\dot{\theta }}_{r}-D\right)-b_{e}{\dot{\theta }}_{e}}{J_{e}}-{\ddot{\theta }}_{\exp}\right)$$

Thus, when considering external disturbances and moment of inertia losses, the robotic system’s control law should be altered as follows:

(28) $$\tau_{m}=\frac{1}{R_{1}R_{2}}\left(J_{e}\left(-\tilde{\alpha }s-\tilde{\beta }\mathrm{sgn}{\left(s\right)}^{\rho }+{\ddot{\theta }}_{\exp}-\frac{\alpha \dot{e}}{\beta +\chi \gamma {\left|\dot{e}\right|}^{\gamma -1}}\right)+b_{e}{\dot{\theta }}_{e}\right)+J_{eq}{\ddot{\theta }}_{r}+b_{eq}{\dot{\theta }}_{r}$$

Substituting (28) into (27), yields the expression:

(29) $$\begin{array}{ll} \dot{s} & =\alpha \dot{e}+\left(\beta +\chi \gamma \mathrm{sgn}{\left(\dot{e}\right)}^{\gamma -1}\right)\ddot{e}\\ & =\alpha \dot{e}+\left(\beta +\chi \gamma \mathrm{sgn}{\left(\dot{e}\right)}^{\gamma -1}\right)\left(\begin{array}{l} -\tilde{\alpha }s-\tilde{\beta }\mathrm{sgn}{\left(s\right)}^{\rho }-\\ \frac{R_{1}R_{2}D}{J_{e}}-\frac{\alpha \dot{e}}{\beta +\chi \gamma {\left|\dot{e}\right|}^{\gamma -1}} \end{array}\right)\\ & =\left(-\tilde{\alpha }s-\tilde{\beta }\mathrm{sgn}{\left(s\right)}^{\rho }-\frac{R_{1}R_{2}D}{J_{e}}\right)\left(\beta +\chi \gamma {\left|\dot{e}\right|}^{\gamma -1}\right) \end{array}$$

By then expanding Equation (29), one can acquire the following formula:

(30) $$\begin{array}{ll} \dot{s} & =-\tilde{\alpha }\left(\beta +\chi \gamma \mathrm{sgn}{\left(\dot{e}\right)}^{\gamma -1}\right)s-\tilde{\beta }\left(\beta +\chi \gamma \mathrm{sgn}{\left(\dot{e}\right)}^{\gamma -1}\right)\mathrm{sgn}{\left(s\right)}^{\rho }-\frac{R_{1}R_{2}D\left(\beta +\chi \gamma \mathrm{sgn}{\left(\dot{e}\right)}^{\gamma -1}\right)}{J_{e}}\\ & =-\tilde{\alpha }\left(\beta +\chi \gamma \mathrm{sgn}{\left(\dot{e}\right)}^{\gamma -1}\right)s-\frac{R_{1}R_{2}D\left(\beta +\chi \gamma \mathrm{sgn}{\left(\dot{e}\right)}^{\gamma -1}\right)}{J_{e}s}s-\tilde{\beta }\left(\beta +\chi \gamma \mathrm{sgn}{\left(\dot{e}\right)}^{\gamma -1}\right)\mathrm{sgn}{\left(s\right)}^{\rho }\\ & =-\left[\tilde{\alpha }\left(\beta +\chi \gamma \mathrm{sgn}{\left(\dot{e}\right)}^{\gamma -1}\right)+\frac{R_{1}R_{2}D\left(\beta +\chi \gamma \mathrm{sgn}{\left(\dot{e}\right)}^{\gamma -1}\right)}{J_{e}s}\right]s-\tilde{\beta }\left(\beta +\chi \gamma \mathrm{sgn}{\left(\dot{e}\right)}^{\gamma -1}\right)\mathrm{sgn}{\left(s\right)}^{\rho } \end{array}$$

For the previously described scenario, in which moment of inertia variations and external disturbances are assumed, it can be known that s and *D* satisfy bounded convergence. After changing the parameters, Equation (30) can be rewritten in the following form:

(31) $$\dot{s}=-{\tilde{\alpha }}^{\prime }s-{\tilde{\beta }}^{\prime }\mathrm{sgn}{\left(s\right)}^{\rho }$$

where ${\tilde{\alpha }}^{\prime }$ and ${\tilde{\beta }}^{\prime }$ are the parameters of the reaching law that satisfies the condition in the case of interference. Hence, for the interference case, the Lyapunov derivative Equation may be represented as follows:

(32) $$\begin{array}{ll} \dot{V} & =s\dot{s}=s\left(-{\tilde{\alpha }}^{\prime }s-{\tilde{\beta }}^{\prime }\mathrm{sgn}{\left(s\right)}^{\rho }\right)\\ & =-{\tilde{\alpha }}^{\prime }s^{2}-{\tilde{\beta }}^{\prime }\mathrm{sgn}{\left(s\right)}^{\rho }s=-2{\tilde{\alpha }}^{\prime }V-2{\tilde{\beta }}^{\prime }{\left|V\right|}^{\frac{1+\rho }{2}}\leq 0 \end{array}$$

thereby satisfying the condition:

(33) $$\dot{V}+2\tilde{\beta }{\left|V\right|}^{\frac{1+\rho }{2}}+2\tilde{\alpha }V\leq 0$$

Thus, when the system is subjected to small changes in the moment of inertia and external disturbance values, the Lyapunov derivative Equation continues to be negative semidefinite, which in accordance with the lemma yields:

(34) $$T_{finite-time}\leq \frac{1}{2{\tilde{\beta }}^{\prime }\left(1-\frac{1+\rho }{2}\right)}\ln\frac{2{\tilde{\beta }}^{\prime }V^{1-\frac{1+\rho }{2}}\left(x_{o}\right)+2{\tilde{\alpha }}^{\prime }}{2{\tilde{\alpha }}^{\prime }}$$

Therefore, considering minimal variation of the moment of inertia and damping values, the energy loss term, and the external disturbance caused by deformation of the nonlinear elastic component, the designed compliant robot trajectory tracking terminal sliding mode controller, under the given initial conditions, can still converge within a finite time, thereby proving the system’s robustness.

## 5. Experimental Results

In this section, the proposed control algorithm is tested on the robot prototype to evaluate its trajectory controlling performance. The control scheme’s performance is then compared to that of a sensor-based, PD, trajectory-tracking controller, in order to prove feasibility and practical stability of the resulting closed-loop system, under the same experimental conditions.

### 5.1. Experimental Setup

**Figure 4:**
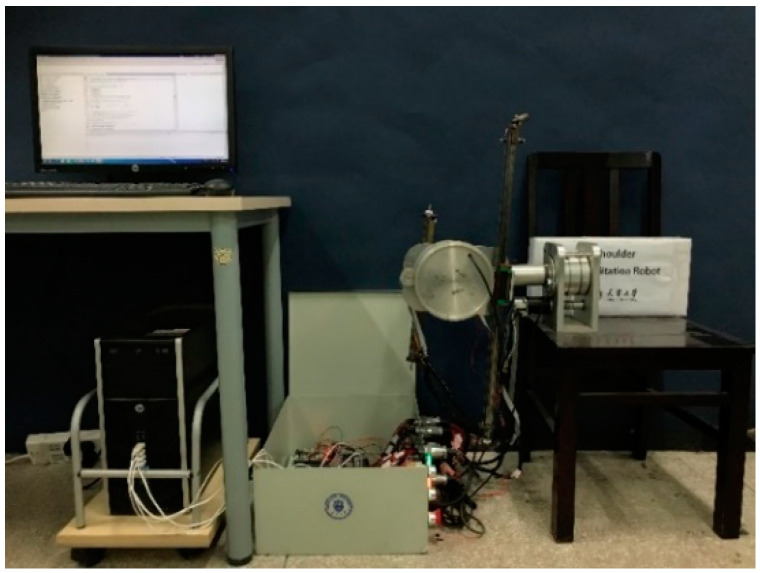
The entire experimental platform.

The prototype of a compliant robot with nonlinear stiffness joints has been developed in our group which has three actuated DOFs shown in Figure 4. The HP host computer based on 64 bit windows-7.1 with Intel Core i7 processor @ 2.40 GHz and 8 GB ram is used to run the control algorithm. The CCS software is used to run the C language program code. The execution rate of the control algorithm is up to 1 kHz, which meets the requirements of the system to process data acquisition and control commands. The DSP board of TMS320F28335 produced by TI company is used to read and process the signal from the maxon motor encoder and transmit it to the host computer. The motor drive adopts an ESCON motor driver. In this paper, an ad electromagnetic tracking system (model: tradstar, produced by NDI company) is used to obtain the position of the end of the robot.

### 5.2. Comparison Experiments in Single Joint

**Figure 5:**
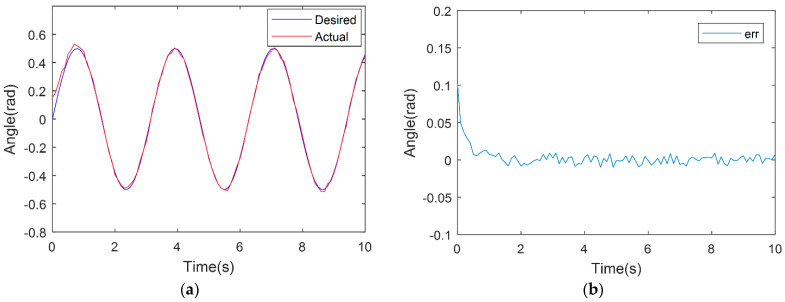
PD-based controller experimental result. (**a**) PD sinusoidal trajectory-tracking experiment results. (**b**) PD sinusoidal tracking experimental errors.

**Figure 6:**
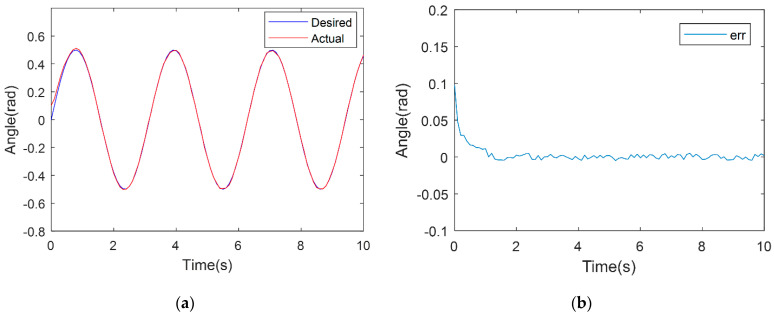
TSM-based controller experimental result. (**a**) TSM sinusoidal trajectory tracking experiment results. (**b**) TSM sinusoidal tracking experimental error value.

In order to verify the control system’s feasibility, a trajectory tracking experiment is firstly carried out using a single joint, thereby attesting to the designed terminal sliding-mode trajectory tracking control system’s practicability. On this basis, the experiments are extended to the trajectory tracking control of the three-joint compliant robot’s end-effector. The desired trajectory is set as $\theta_{\exp}=0.5\sin(2t)$. To ensure fairness of the experiment and validity of the comparative analysis, experiments were performed using the same prototype, within the same condition, and the parameters of the PD controller were optimized. The trajectory tracking results of the compared TSM method and PD controller are shown in Figure 5 and Figure 6. From Figure 5, the PD controller is also able to obtain a good performance of tracking a sinusoidal signal and the stabilized error is lower than 0.002 rad, even when strong nonlinear factors exist in this prototype. In Figure 6, TSM shows a better performance and the stabilized error is about 0.001 rad. The single-joint experiment results reveal that under identical conditions, trajectory tracking of the TSM controller designed in this paper exhibits heightened trajectory tracking performance, with smaller tracking error values and faster convergence times, in comparison to the PD controller because the nonlinear factor exists in a single joint introduced by friction and hysteresis.

### 5.3. Comparison Experiments on the Prototype of 3 DoF Compliant Robot

**Figure 7:**
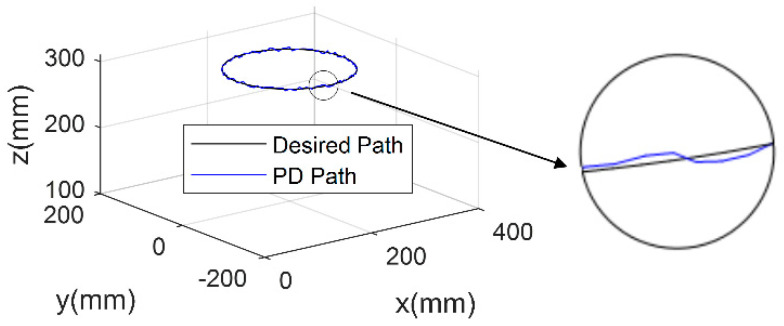
PD-based trajectory tracking controller experimental result.

**Figure 8:**
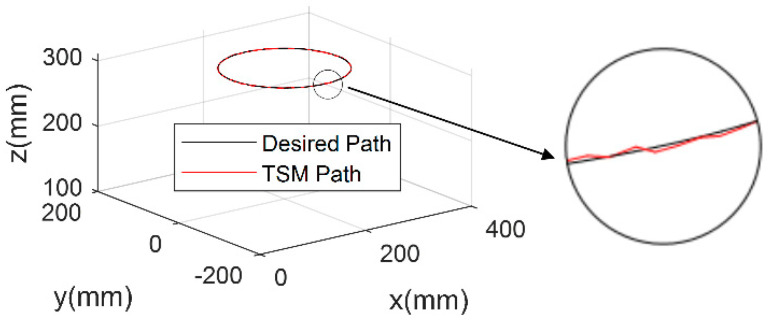
TSM-based trajectory tracking controller experimental result.

Analyzing the single-joint, trajectory-tracking experiment results leads to the corroboration of the proposed terminal sliding mode trajectory control system’s feasibility. In order to further test the efficacy of the TSM method to deal with the robot with multiple NSAs, a compliant robot end-effector trajectory-tracking experiment is performed. The designed referential trajectory entails a circular path in Cartesian space. This setting can cause significant joint parameter variations, and therefore provide a thorough controller performance validation. In order to make the experiments convincing, the PD controller adopts optimized parameters to obtain the best parameters, and the pertinent comparative test results are displayed in Figure 7 and Figure 8.

To further analyze the experimental data, the root mean square error is defined as follows:

(35) $$RMSE=\sqrt{\left(\sum_{t=0}^{T}{\left\|\xi_{e}(t)-\xi_{\exp}(t)\right\|}_{2}\right)/T}$$

**Figure 9:**
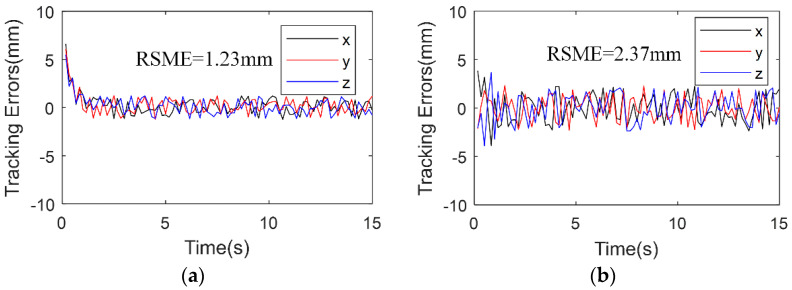
Trajectory tracking variance mean square. (**a**) RMSE of TSM-based controller trajectory tracking. (**b**) RMSE of Trajectory tracking based on PD controller.

The analysis results of tracking errors along three axes are shown in Figure 9. It can be seen from these results that the tracking errors corresponding to the terminal sliding-mode trajectory tracking controller is rather small. Moreover, it is observed that the proposed controller outperforms the traditional PD controller, in terms of trajectory-tracking accuracy, since its root mean square error value is 1.23 mm, which is smaller than the 2.37 mm value pertaining to the PD controller.

**Figure 10:**
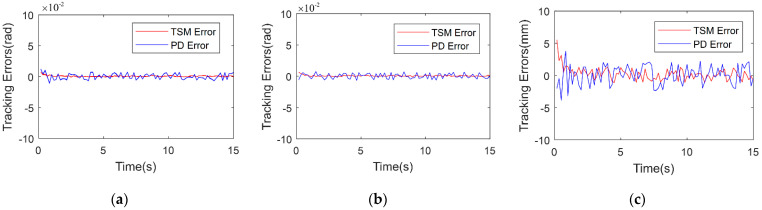
Trajectory tracking error of joints. (**a**) The first joint tracking error. (**b**) The second joint tracking error. (**c**) The third joint tracking error.

In Figure 10, the tracking errors in every joint which has nonlinear stiffness property are given. The mean tracking errors of the first and the second are lower than 0.002 rad in both the PD controller and TSM controller, and it is obvious that the errors in the TSM controller is lower than the PD controller in rotational joint with nonlinear stiffness. For the third joint, in fact it is a translational joint which is driven by a cable-pulley mechanism, and its errors are larger than those in the other two joints because the driven structure is different where the cable connects two positions with a long distance in the third joint, which introduces vibration and flab. However, the mean errors of the TSM controller is also lower than those of the PD controller, which means the proposed method is also an effect in translational joint.

## 6. Conclusions

In the NSA, due to the imperfect performance of the elastic mechanical component, it is more difficult to achieve accurate position control compared to the rigid actuator. Moreover, for a compliant robot with multiple degree of freedoms (DOFs) driven by NSAs, the influence of every NSA on the trajectory of the end effector is different and even coupled. To perform accurate trajectory-tracking of the end effector on a compliant robot, a controller based on terminal sliding mode is designed. A stability proof is derived based on Lyapunov theory, which considers the application of the proposed control scheme onto the presented dynamical model. Moreover, the resulting closed-loop system’s time convergence and its robustness are verified. The single-joint sinusoidal trajectory experiment, and the end-effector planar prototype trajectory experiment, are carried out under the same experimental conditions on a compliant 3-DoF robot with NSAs developed in our team, while the PD parameters are assigned with optimal values. These experiments verify the effectiveness and superiority of the proposed algorithm. In the future, the proposed algorithm will be applicable in compliant robots including industrial robots, wearable robots and exoskeletons.

## References

[1] Calanca A., Muradore R., Fiorini P. (2015). A Review of Algorithms for Compliant Control of Stiff and Fixed-Compliance Robots. IEEE/ASME Trans. Mechatron..

[2] Paine N., Oh S., Sentis L. (2013). Design and Control Considerations for High-Performance Series Elastic Actuators. IEEE/ASME Trans. Mechatron..

[3] Sun J., Zhang Y., Zhang C., Guo Z., Xiao X. Mechanical design of a compact Serial Variable Stiffness Actuator (SVSA) based on lever mechanism. Proceedings of the 2017 IEEE International Conference on Robotics and Automation (ICRA).

[4] Song Z., Lan S., Dai J.S. (2019). A New Mechanical Design Method of Compliant Actuators with Non-linear Stiffness with Predefined Deflection-torque Profiles. Mech. Mach. Theory.

[5] Zhao Y., Song Z., Ma T., Dai J.S. (2020). Optimisation of Stiffness to Achieve Increased Bandwidth and Torque Resolution in Nonlinear Stiffness Actuators. IEEE Trans. Ind. Electron..

[6] Hogan N. (1985). Impedance Control: An Approach to Manipulation: Part I—Theory. Trans. ASME J. Dyn. Syst. Meas. Control.

[7] Hurst J.W., Chestnutt J.E., Rizzi A. An Actuator with Physically Variable Stiffness for Highly Dynamic Legged Locomotion. Proceedings of the IEEE International Conference on Robotics and Automation, ICRA 2004.

[8] Erler P., Beckerle P., Strah B., Rinderknecht S. Experimental Comparison of Nonlinear Motion Control Methods for a Variable Stiffness Actuator. Proceedings of the 5th IEEE RAS/EMBS International Conference on Biomedical Robotics and Biomechatronics.

[9] Ramirez-Neria M., Ochoa-Ortega G., Lozada-Castillo N., Trujano-Cabrera M.A., Campos-Lopez J.P., Luviano-Juárez A. (2016). On the Robust Trajectory Tracking Task for Flexible-joint Robotic Arm with Unmodeled Dynamics. IEEE Access.

[10] Han J. (2009). From PID to Active Disturbance Rejection Control. IEEE Trans. Ind. Electron..

[11] Spong W. (1987). Modeling and Control of Elastic Joint Robots. ASME J. Dyn. Syst. Meas. Control.

[12] Jamwal P.K., Hussain S., Ghayesh M.H., Rogozina S.V. (2016). Impedance Control of an Intrinsically Compliant Parallel Ankle Rehabilitation Robot. IEEE Trans. Ind. Electron..

[13] Albu-schäffer A., Ott C., Hirzinger G. (2007). A Unified Passivity-based Control Framework for Position, Torque and Impedance Control of Flexible Joint Robots. Int. J. Robot. Res..

[14] Sira-ramirez H., Spong W. (1988). Variable Structure Control of Flexible Joint Manipulator. Int. J. Robot. Autom..

[15] Zeman V., Patel R., Khorasani K. (1997). Control of Flexible-joint Robot Using Neural Networks. IEEE Trans. Control Syst. Technol..

[16] Zheng D., Pan Y., Guo K., Yu H. (2019). Identification and Control of Nonlinear Systems Using Neural Networks: A Singularity-Free Approach. IEEE Trans. Neural Netw. Learn. Syst..

[17] Pan Y., Wang H., Li X., Yu H. (2018). Adaptive Command-Filtered Backstepping Control of Robot Arms with Compliant Actuators. IEEE Trans. Control Syst. Technol..

[18] Su H., Hu Y., Karimi H.R., Knoll A., Ferrigno G., De Momi E. (2020). Improved recurrent neural network-based manipulator control with remote center of motion constraints: Experimental results. Neural Netw..

[19] Su H., Qi W., Hu Y., Karimi H.R., Ferrigno G., De Momi E. (2020). An Incremental Learning Framework for Human-like Redundancy Optimization of Anthropomorphic Manipulators. IEEE Trans. Ind. Inform..

[20] Luo J., Huang D., Li Y., Yang C. (2021). Trajectory Online Adaption Based on Human Motion Prediction for Teleoperation. IEEE Trans. Autom. Sci. Eng..

[21] Luo J., Lin Z., Li Y., Yang C. (2020). A Teleoperation Framework for Mobile Robots Based on Shared Control. IEEE Robot. Autom. Lett..

[22] Cao Y., Xiang K., Tang B., Ju Z., Pang M. (2019). Design of Muscle Reflex Control for Human Upright Standing Push- recovery based on Series Elastic Actuator. Proceedings of the 2019 IEEE 9th Annual International Conference on CYBER Technology in Automation, Control, and Intelligent Systems (CYBER).

[23] Nakao M., Ohnishi K., Miyachi K. (1987). A Robust Decentralized Joint Control based on Interference estimation. Proceedings of the IEEE International Conference on Robotics and Automation.

[24] Yang T., Sun N., Fang Y., Xin X., Chen H. (2021). New Adaptive Control Methods for n-Link Robot Manipulators with Online Gravity Compensation: Design and Experiments. IEEE Trans. Ind. Electron..

[25] Lee T., Kwon J., Park F.C. A Natural Adaptive Control Law for Robot Manipulators. Proceedings of the 2018 IEEE/RSJ International Conference on Intelligent Robots and Systems (IROS).

[26] Wu L., Yan Q., Cai J. (2019). Neural Network-Based Adaptive Learning Control for Robot Manipulators with Arbitrary Initial Errors. IEEE Access.

[27] Jung S. (2018). Improvement of Tracking Control of a Sliding Mode Controller for Robot Manipulators by a Neural Network. Int. J. Control Autom. Syst..

[28] Sun C., Gao H., He W., Yu Y. (2018). Fuzzy Neural Network Control of a Flexible Robotic Manipulator Using Assumed Mode Method. IEEE Trans. Neural Netw. Learn. Syst..

[29] Alattas K.A., Mobayen S., Din S.U., Asad J.H., Fekih A., Assawinchaichote W., Vu M.T. (2021). Design of a Non-Singular Adaptive Integral-Type Finite Time Tracking Control for Nonlinear Systems with External Disturbances. IEEE Access.

[30] Thanh H.L.N.N., Mung N.X., Nguyen N.P., Phuong N.T. (2020). Perturbation observer-based robust control using a multiple sliding surfaces for nonlinear systems with influences of matched and unmatched uncertainties. Mathematics.

[31] Yu S., Yu X., Shirinzadeh B., Man Z. (2005). Continuous Finite-time Control for Robotic Manipulators with Terminal Sliding Mode. Automatica.

[32] Lan S., Song Z. (2016). Design of a New Nonlinear Stiffness Compliant Actuator and its Error Compensation Method. J. Robot..

